# The elimination of human African trypanosomiasis: Monitoring progress towards the 2021–2030 WHO road map targets

**DOI:** 10.1371/journal.pntd.0012111

**Published:** 2024-04-16

**Authors:** Jose R. Franco, Gerardo Priotto, Massimo Paone, Giuliano Cecchi, Agustin Kadima Ebeja, Pere P. Simarro, Dieudonne Sankara, Samia B. A. Metwally, Daniel Dagne Argaw

**Affiliations:** 1 World Health Organization, Global Neglected Tropical Diseases Programme, Prevention, Treatment and Care Unit, Geneva, Switzerland; 2 Food and Agriculture Organization of the United Nations, Animal Production and Health Division, Rome, Italy; 3 World Health Organization, Regional Office for Africa, Communicable Disease Unit, Brazzaville, Congo; 4 Consultant, World Health Organization, Global Neglected Tropical Diseases Programme, Innovative and Intensified Disease Management Unit, Geneva, Switzerland; Keele University, UNITED KINGDOM

## Abstract

**Background:**

Human African trypanosomiasis (HAT) is a neglected tropical disease that usually occurs in rural areas in sub-Saharan Africa. It caused devastating epidemics during the 20th century. Sustained, coordinated efforts by different stakeholders working with national sleeping sickness control programmes (NSSCPs) succeeded in controlling the disease and reducing the number of cases to historically low levels. In 2012, WHO targeted the elimination of the disease as a public health problem by 2020. This goal has been reached and a new ambitious target was stated in the WHO road map for NTDs 2021–2030 and endorsed by the 73rd World Health Assembly: the elimination of gambiense HAT transmission (i.e. reducing the number of reported cases to zero). The interruption of transmission was not considered as an achievable goal for rhodesiense HAT, as it would require vast veterinary interventions rather than actions at the public health level.

**Methodology/principal findings:**

Data reported to WHO by NSSCPs were harmonized, verified, georeferenced and included in the atlas of HAT. A total of 802 cases were reported in 2021 and 837 in 2022. This is below the target for elimination as a public health problem at the global level (< 2000 HAT cases/year); 94% of the cases were caused by infection with *T*. *b*. *gambiense*. The areas reporting ≥ 1 HAT case/10 000 inhabitants/year in 2018–2022 cover a surface of 73 134 km^2^, with only 3013 km^2^ at very high or high risk. This represents a reduction of 90% from the baseline figure for 2000–2004, the target set for the elimination of HAT as a public health problem. For the surveillance of the disease, 4.5 million people were screened for gambiense HAT with serological tests in 2021–2022, 3.6 million through active screening and 0.9 million by passive screening.

In 2021 and 2022 the elimination of HAT as a public health problem was validated in Benin, Uganda, Equatorial Guinea and Ghana for gambiense HAT and in Rwanda for rhodesiense HAT. To reach the next goal of elimination of transmission of gambiense HAT, countries have to report zero cases of human infection with *T*. *b*. *gambiense* for a period of at least 5 consecutive years. The criteria and procedures to verify elimination of transmission have been recently published by WHO.

**Conclusions/significance:**

HAT elimination as a public health problem has been reached at global level, with seven countries already validated as having reached this goal. This achievement was made possible by the work of NSSCPs, supported by different public and private partners, and coordinated by WHO. The new challenging goal now is to reach zero cases by 2030. To reach this goal is crucial to maintain the engagement and support of donors and stakeholders and to keep the involvement and coordination of all partners.

Along with the focus on elimination of transmission of gambiense HAT, it is important not to neglect rhodesiense HAT, which is targeted for elimination as a public health problem in the WHO road map for NTDs 2021–2030.

## Author summary

Human African trypanosomiasis (HAT), also known as “sleeping sickness, is a neglected tropical disease (NTD) transmitted by the bite of infected tsetse flies mainly affecting rural areas in sub-Saharan Africa. During the 20th century it caused enormous suffering in the endemic areas with a last reported peak in the late 1990s. This situation triggered a successful coordinated control effort that allowed, in 2012, the targeting of elimination of the disease as a public health problem by 2020. This paper follows previous papers to present the achievements towards HAT elimination. Annually reported cases have been kept below 1000 cases per year. HAT surveillance is maintained through active screening in endemic areas but also within a network of health facilities able to detect the disease. The World Health Organization (WHO), as defined in its 2021–2030 road map for NTDs, is now targeting the elimination of transmission of gambiense HAT and the elimination of rhodesiense HAT as a public health problem. The commitment of national health authorities and the international community will be essential to achieve these ambitious targets.

## Introduction

Human African trypanosomiasis (HAT), or sleeping sickness, is a parasitic disease caused by infection with either of two subspecies of *Trypanosoma brucei* (i.e. *T*. *b*. *gambiense* and *T*. *b*. *rhodesiense*), flagellate protozoa belonging to the Kinetoplastida class [1,2].

The disease is transmitted in sub-Saharan Africa, within the distributional limits of its vector, the tsetse fly (genus: *Glossina*). The disease usually occurs in rural areas, and it is characterized by a markedly focal distribution related to the complex interactions between parasite, vector, hosts, and the environment [3,4]. HAT mainly affects populations engaged in activities that expose them to frequent contacts with tsetse [5]. These populations are usually endowed with scarce economic resources, and they are often served by weak health care systems. These features and the limited investment in research and control place HAT among the neglected tropical diseases (NTDs) [6].

Of the two existing forms of HAT, the slower-progressing form is caused by infection with *T*. *b*. *gambiense*, and it is endemic in western and central Africa; the faster progressing form, caused by infection with *T*. *b*. *rhodesiense*, is found in eastern and southern Africa [2,5,7]. HAT cases can also be detected outside the endemic countries among travellers, tourists and migrants having visited or coming from sub-Saharan Africa [8,9]. The gambiense form is responsible for the vast majority of reported cases [10,11], and it is considered mainly anthroponotic with humans as the main reservoir. Domestic and wild animals can also host *T*. *b*. *gambiense*, although their epidemiological role appears to be limited [12]. Conversely, livestock and wildlife are the main reservoirs of *T*. *b*. *rhodesiense*, and therefore this form of HAT is considered a zoonosis [13,14]. Sleeping sickness almost invariably progresses to death unless it is correctly diagnosed and treated [15].

HAT caused devastating epidemics during the 20th century [16,17], with the last peak of infections at the turn of the century (i.e. more than 35 000 cases reported annually in 1997 and 1998) [13]. The sustained and coordinated efforts of a broad range of stakeholders (pharmaceutical companies, bilateral cooperation agencies, nongovernmental organizations (NGOs), research institutions and philanthropic organizations), working together with the National Sleeping Sickness Control Programmes (NSSCPs) in endemic countries [18,19], succeeded in reversing the epidemiological trend, controlling the disease and reducing the number of cases to historically low levels [20]. In particular, in 2012 WHO targeted the elimination of the disease as a public health problem by 2020. This goal was included in the first WHO road map for NTDs [6], endorsed in 2013 by the 66th World Health Assembly [21]. Specifically, the targets were defined as “fewer than 2,000 HAT cases reported per year”, and “a 90% reduction in the areas at moderate or higher risk compared to the 2000–2004 baseline” [10]. Biennial reports monitoring progress towards these targets are available from 2012 to 2020 [10,20,22–24], and they show that, as of 2017, fewer than 2000 cases/year were reported globally, and that in 2016–2020 the reduction of the areas at moderate or higher risk was 83% as compared with the 2000–2004 baseline [20].

Against this backdrop, a new challenging target was set for 2030, i.e. the elimination of gambiense HAT transmission by reducing the number of reported cases to zero. This goal is stated in the subsequent road map for NTDs 2021–2030 [25], which was officially endorsed in 2020 by the 73rd World Health Assembly (WHA 73.33) [26]. In this road map the interruption of transmission was not considered as an achievable goal for rhodesiense HAT, mainly because its zoonotic nature would require vast veterinary interventions rather than actions at the public health level.

The present paper follows up on previous reports, and it presents an update of the HAT situation with a focus on 2021–2022 and the progress towards the targets set in the WHO road map for NTDs 2021–2030 (“the road map”).

## Materials and methods

### Ethics statement

This paper does not involve research with human participants. No individual data is used. All the data used are provided as routine epidemiological information and are fully anonymized before transmission.

### Indicators and targets

The key global indicators used to monitor the progress towards gambiense HAT elimination of transmission are provided by the road map [27], and they include: (i) the number of cases reported annually; and (ii) the number of countries verified for elimination of transmission, where “verification” is the process of documenting the elimination of disease transmission at the country level [28]. For these two key indicators, quantitative targets are set in the road map, namely zero HAT cases reported, and 15 countries verified for interruption of transmission. Other indicators that are monitored are: (i) area at risk (reporting ≥ 1 case/10 000 people/year), (ii) population at risk (population living in the areas at risk); (iii) coverage of the at-risk populations by control and surveillance activities; (iv) number of countries validated for elimination as a public health problem. For these secondary indicators no specific target is set.

For rhodesiense HAT, the key global indicators used to monitor its elimination as a public health problem according to the road map (i.e. for which quantitative targets apply) are (i) the number of countries validated for elimination as a public health problem, where “validation” is the process of documenting elimination as a public health problem at the country level, and (ii) the area at risk (reporting ≥ 1 case/10 000 people/year, calculated over 5-year periods). For these indicators the targets for 2030 are eight countries validated and the areas at moderate risk of rhodesiense HAT (or higher) reduced to zero.

The methodological details used to calculate the indicators were presented in previous dedicated publications [22,29,30].

### Data reporting and analysis

All data presented in this paper were reported to WHO by NSSCPs and their collaborating research institutions. They were collected directly at the field level and, in some cases, they could have limitations in their quality, which varies from country to country. To ensure the best possible quality, data were validated via verification with the sources, harmonized, georeferenced and included in the atlas of HAT [4,31].

### Number of cases and their geographical distribution

HAT cases are reported annually by NSSCPs. The reported figures are subsequently validated by WHO in consultation with the reporting sources [4]. Cases detected in and reported from non-endemic countries are included in the tally of the probable country of infection [8,9]. These data are made publicly available through the WHO Global Health Observatory (www.who.int/data/gho).

Most of the reported cases were confirmed by the observation of the parasite in body fluids or tissues. However, in a few countries some cases were not confirmed by parasitology but, following the WHO recommended case definition [13], they were included in the reported case counts.

For each reported case, the geographical location is recorded in the atlas, based on information provided by the countries. For HAT cases this usually refers to the village of residence of the patient, but it can also refer to the probable place of infection or place of detection when the village of residence is considered highly unsuitable or unlikely for transmission. Exact geographical coordinates are normally provided by the NSSCPs. Alternatively, they are extracted from existing public domain databases or estimated in a Geographic Information System according to the descriptive information provided by NSSCPs [4,31].

#### Area and population at risk

The area and population at risk of HAT are estimated as previously described [29,32]. In a nutshell, risk is estimated as the ratio between the number of reported cases and the exposed populations (using Landscan population distribution data), and both the HAT reported cases and the Landscan population layers are subject to spatial smoothing.

#### Active and passive surveillance activities

Data on active and passive screening activities are provided to WHO by endemic countries. For active and passive screening, the number of people screened and their geographical distribution is collected [23,24].

To assess the coverage of passive surveillance, the geographical distribution of health facilities with capacity for diagnosis of HAT is also reported by NSSCP. This distribution is then used to estimate the potential accessibility to health facilities for populations at risk of HAT [30]. Health facilities with capacity for treatment of HAT are also mapped, and their coverage estimated.

#### Vector control

A description of vector control activities against HAT as implemented by NSSCPs and their partners, including the targeted areas, is included in annual reports to WHO. Efforts are ongoing to enhance the reporting of vector control activities and to harmonize the estimation of its coverage in space and time [33].

### Official validation and verification of HAT elimination at country level

“Validation” of HAT elimination as a public health problem can be claimed by countries reaching (i) the threshold of fewer than 1 case/10 000 inhabitants/year (averaged over the past 5 years) in each health district and (ii) an overall level of control and surveillance activities that can be considered as “adequate” according to its intensity, coverage and effectiveness. For official validation, the Ministry of Health of the claiming country prepares and submits a dossier to WHO. The dossier is evaluated by an independent, ad hoc panel of experts and, based on the outcome of this assessment, WHO can declare the validation of HAT elimination as a public health problem in the country [34]. A similar process will be followed for the verification of elimination of gambiense HAT transmission [35,36].

## Results

### Number of HAT cases reported annually

In the biennium 2021–2022 a total of 1639 HAT cases were reported, of which 1546 were caused by infection with *T*. *b*. *gambiense* (94%). This represents a decrease of 16 cases from the previous biennium (1655 cases in 2019–2020). The number of gambiense HAT cases reported increased by 7.3% (+105 cases) but decreased for rhodesiense HAT by 57% (˗121 cases). For both forms of the disease, 802 cases were reported in 2021 and 837 in 2022 (Tables 1 and 2).

**Table 1:** *T*. *b*. *gambiense* HAT: new cases reported between 2013 and 2022.

Country	2013	2014	2015	2016	2017	2018	2019	2020	2021	2022	Total
Angola	69	36	35	20	18	79	30	33	174	44	538
Benin	0	0	0	0	0	0	0	0	0	0	0
Burkina Faso	0	0	1	0	0	0	0	0	0	0	1
Cameroon	6	7	6	6	5	7	20	2	11	7	77
Central African Republic	59	194	147	101	76	57	86	39	45	110	914
Chad	195	95	67	54	28	12	16	17	15	18	517
Congo	20	21	36	18	15	24	17	15	18	10	194
Côte d’Ivoire	7	6	3	0	3	2	1	0	1	0	23
Democratic Republic of the Congo	5649	3205	2347	1768	1100	660	613	395	424	516	16 677
Equatorial Guinea	3	0	0	3	4	4	3	1	3	13	34
Gabon	17	10	9	10	9	16	8	11	18	21	129
Ghana	1	0	0	0	0	0	0	0	0	0	1
Guinea	78	33	29	108	139	74	69	36	28	30	624
Guinea-Bissau	–	–	–	–	0	–	–	–	–	–	0
Liberia	–	–	–	–	–	–	–	0	0	0	0
Mali	0	0	0	0	0	0	0	0	0	0	0
Niger	–	–	0	–	–	–	–	–	–	–	0
Nigeria	0	0	0	1	0	0	0	0	0	0	1
Senegal	–	–	–	–	–	–	–	–	0	0	0
South Sudan	117	63	45	17	12	17	11	15	10	30	337
Togo	0	0	0	0	0	0	0	0	0	0	0
Uganda	9	9	4	4	0	1	2	1	0	0	30
Total	6230	3679	2729	2110	1409	953	876	565	747	799	20 097

“–” No data reported.

In the Gambia and Sierra Leone no case was reported, but no surveillance activity is known to have been carried out in 2013–2022.

**Table 2:** *T*. *b*. *rhodesiense* HAT: new cases reported between 2013 and 2022.

Country	2013	2014	2015	2016	2017	2018	2019	2020	2021	2022	Total
Ethiopia	–	–	–	–	–	–	–	–	–	6	6
Kenya	0	0	0	0	0	0	0	0	0	0	0
Malawi	35	32	30	35	7	15	91	89	49	24	407
Rwanda	–	–	–	0	0	0	0	0	0	0	0
Uganda	43	70	28	10	13	4	5	2	2	0	177
United Republic of Tanzania	2	1	2	4	3	0	3	1	1	1	18
Zambia	6	12	9	4	3	5	15	6	3	7	70
Zimbabwe	1	3	3	1	1	0	2	0	0	0	11
Total	87	118	72	54	27	24	116	98	55	38	689

“–” No data reported.

Other *T*. *b*. *rhodesiense* HAT historically endemic countries not reporting cases are Burundi and Mozambique, but no surveillance activity is known to have been carried out in 2013–2022. Botswana, Namibia and Eswatini are considered free of the vector responsible for the transmission of *T*. *b*. *rhodesiense* HAT [37,38].

In 2021–2022 a total of 940 cases were reported from the Democratic Republic of the Congo, corresponding to 61% of all gambiense HAT cases. For rhodesiense HAT, 73 cases were reported from Malawi (78% of all rhodesiense cases).

**Fig 1:**
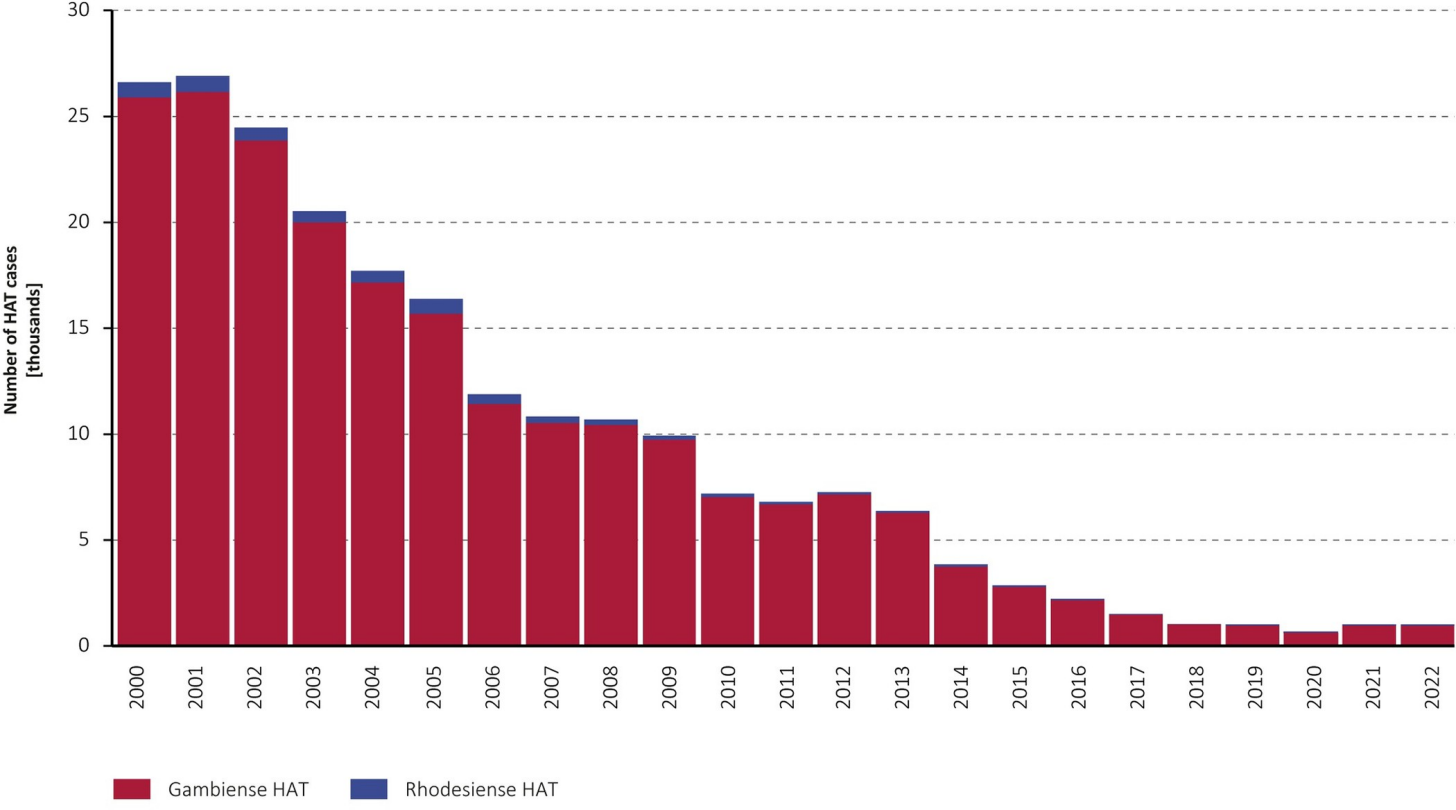
Total number of HAT cases reported annually, 2000–2022.

Four cases of rhodesiense HAT and one case of gambiense HAT were reported from non-endemic countries in 2021–2022. The rhodesiense HAT “exported” cases were detected in South Africa, the United States of America (USA), Germany and Denmark, and they were estimated to have been infected in Zambia (3) and Malawi (1). The gambiense HAT “exported” case was diagnosed in Kenya (country considered as endemic for rhodesiense HAT but not for gambiense HAT) in a patient coming from South Sudan. All these cases are included in the counts in Tables 1 and 2, which show the number of gambiense and rhodesiense HAT cases reported by endemic countries during 2013–2022. Fig 1 shows the overall trend in reported HAT cases since 2000.

### Geographical distribution of HAT cases

**Fig 2:**
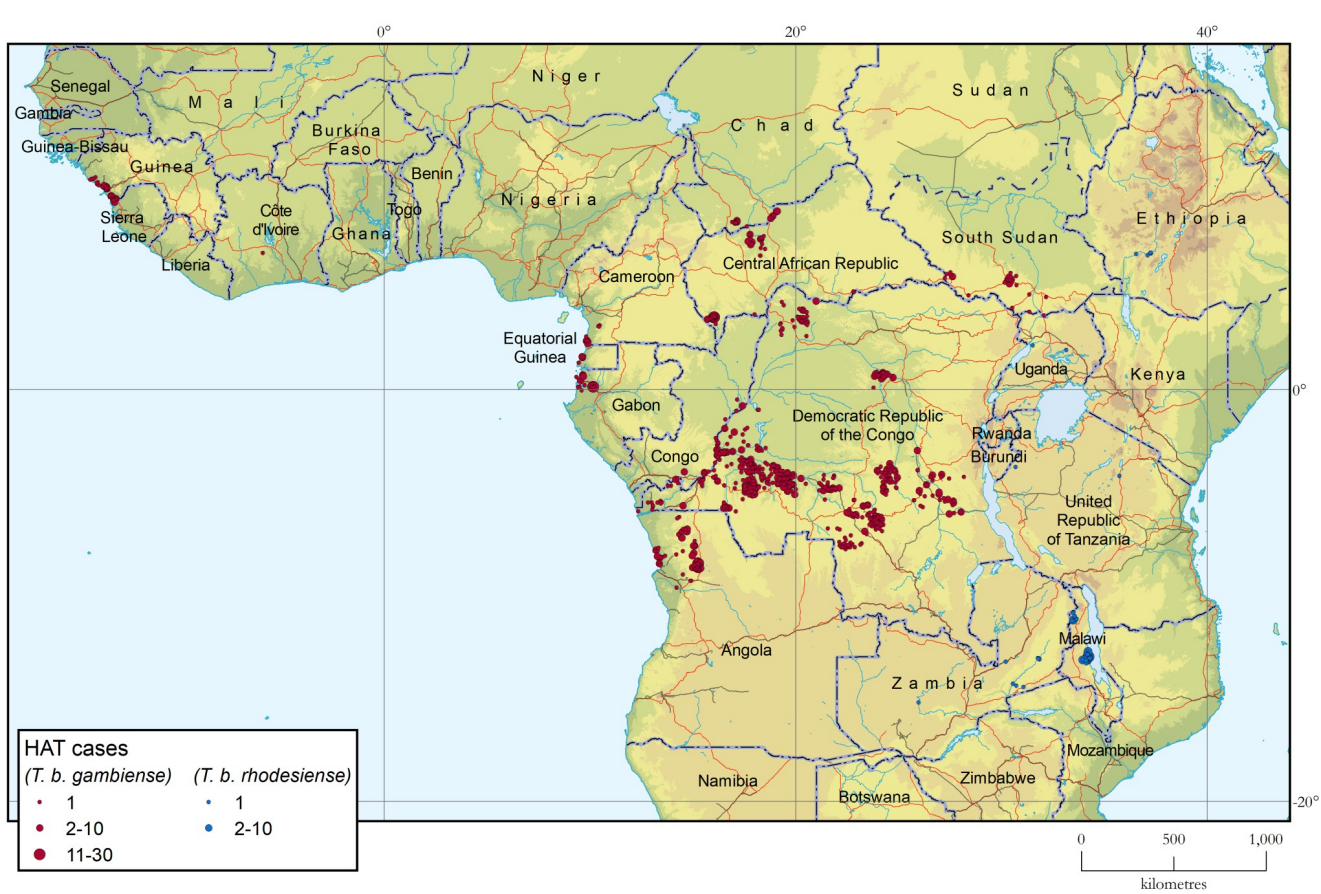
Geographical distribution of HAT, 2021–2022. The base layers used in this map are the FAO Global Administrative Unit Layers (GAUL) https://data.apps.fao.org/map/catalog/srv/eng/catalog.search#/metadata/9c35ba10-5649-41c8-bdfc-eb78e9e65654, Shuttle Radar Topography Mission (SRTM) https://doi.org/10.5066/F7F76B1X, FAO Inland water bodies in Africa https://data.apps.fao.org/map/catalog/srv/eng/catalog.search;jsessionid=B7AF7A215B16770A1A67C65D97FF21CA?node=srv#/metadata/bd8def30-88fd-11da-a88f-000d939bc5d8, FAO Rivers of Africa https://data.apps.fao.org/map/catalog/srv/eng/catalog.search;jsessionid=B7AF7A215B16770A1A67C65D97FF21CA?node=srv#/metadata/b891ca64-4cd4-4efd-a7ca-b386e98d52e8 and Vector Map Level 0 (VMap0) https://gis-lab.info/qa/vmap0-eng.html.

Fig 2. shows the geographical distribution of sleeping sickness cases in Africa in 2021–2022 (S1 Fig provides a larger-scale subregional maps).

#### Gambiense HAT

In western Africa 59 HAT cases were reported in 2021–2022. All of these cases were restricted to the coastal region of Guinea, except for one that was detected in Côte d’Ivoire. For the latter there is strong evidence that infection may have occurred before 2021, as the patient was already a serological suspect in 2020, and was just confirmed parasitologically in 2022 during follow-up. In Guinea the decreasing trend observed after the post-Ebola upshot in 2016–2017 [39] is continuing. In Côte d’Ivoire the trend towards a sustained zero reported cases situation is clear [40]. No cases were reported in this biennium in the other countries of the subregion. Adequate surveillance is in place in Benin, Burkina Faso, Ghana, Liberia, Mali, Senegal and Togo, while during the same period no, or very limited, HAT surveillance and control activities were carried out in the Gambia, Guinea-Bissau, Sierra Leone, Niger and Nigeria.

In the central Africa region, the intensity and coverage of HAT surveillance and control activities remained fairly stable, as in previous years. Important challenges nevertheless persist in terms of the availability of resources and, in some instances, security (i.e. South Sudan and Central African Republic). Chad and Congo reported a stable number of cases in the past 2 years (between 10 and 20 cases yearly), and a similarly stable situation was observed in Cameroon, regardless of the very low number of cases reported in 2020 from this country as a result of low HAT surveillance in that year. In Gabon, the number of cases increased in the last biennium, following intensified control and surveillance activities. In the Central African Republic, despite security constraints still affecting certain areas, the general situation improved and activities were reinforced with an important increase in the number of cases reported in 2022. In Equatorial Guinea the number of cases had been below 5 cases per annum for more than 10 years, but an unexpected increase was reported in 2022 in the Mbini focus (11 cases).

In Angola a surge in cases was reported. This was linked to the reinforcement of passive and active screening activities. However, some concerns about the quality of the diagnosis have been raised, which warrant further scrutiny.

A slight increase in the number of gambiense HAT cases has been reported in the Democratic Republic of the Congo. This followed a notable decrease in 2020, which was mainly ascribed to decreased surveillance activities during the coronavirus disease (COVID-19) pandemic. With 424 cases in 2021 and 516 in 2022 it remains the country with the highest number of cases reported.

No gambiense HAT cases were reported in Uganda in 2021–2022, despite intense surveillance activities in the West Nile region. Interestingly, however, targeted vector control was maintained in the area [41]. In South Sudan the number of reported cases increased in 2022, following the reinforcement of active and passive screening activities that was made possible by an improved security situation; however, some concerns exist about the quality and capacity of diagnostic capability in this setting.

#### Rhodesiense HAT

Malawi remains the country reporting the most cases of rhodesiense HAT (78%). However, after the outbreak in 2019–2020, the number of cases abated, and figures returned to the level of previous years. Cases of rhodesiense HAT also occurred in Zambia (3 in 2021 and 7 in 2022), with fewer sporadic cases reported from the United Republic of Tanzania and Uganda. No cases were declared in the 2021–2022 biennium in Kenya, Rwanda and Zimbabwe, where an adequate surveillance system is in place. The majority of rhodesiense HAT cases declared in recent years occurred in people interacting with protected areas, where the wildlife reservoir is considered a main source of disease transmission.

The report of 6 cases of rhodesiense HAT in Ethiopia in 2022 is noteworthy, as no case had been reported from this country since 1991 [42,43]. These new cases were detected in the Southern Nations, Nationalities, and Peoples’ Region (SNNPR); more specifically, they were infected in Kucha Alfa, Denba Gofa and Melekoza districts (Gamo and Goffa zones respectively) [44]. These areas are known to be endemic for bovine trypanosomiasis [45].

### Areas and population at risk of HAT

**Fig 3:**
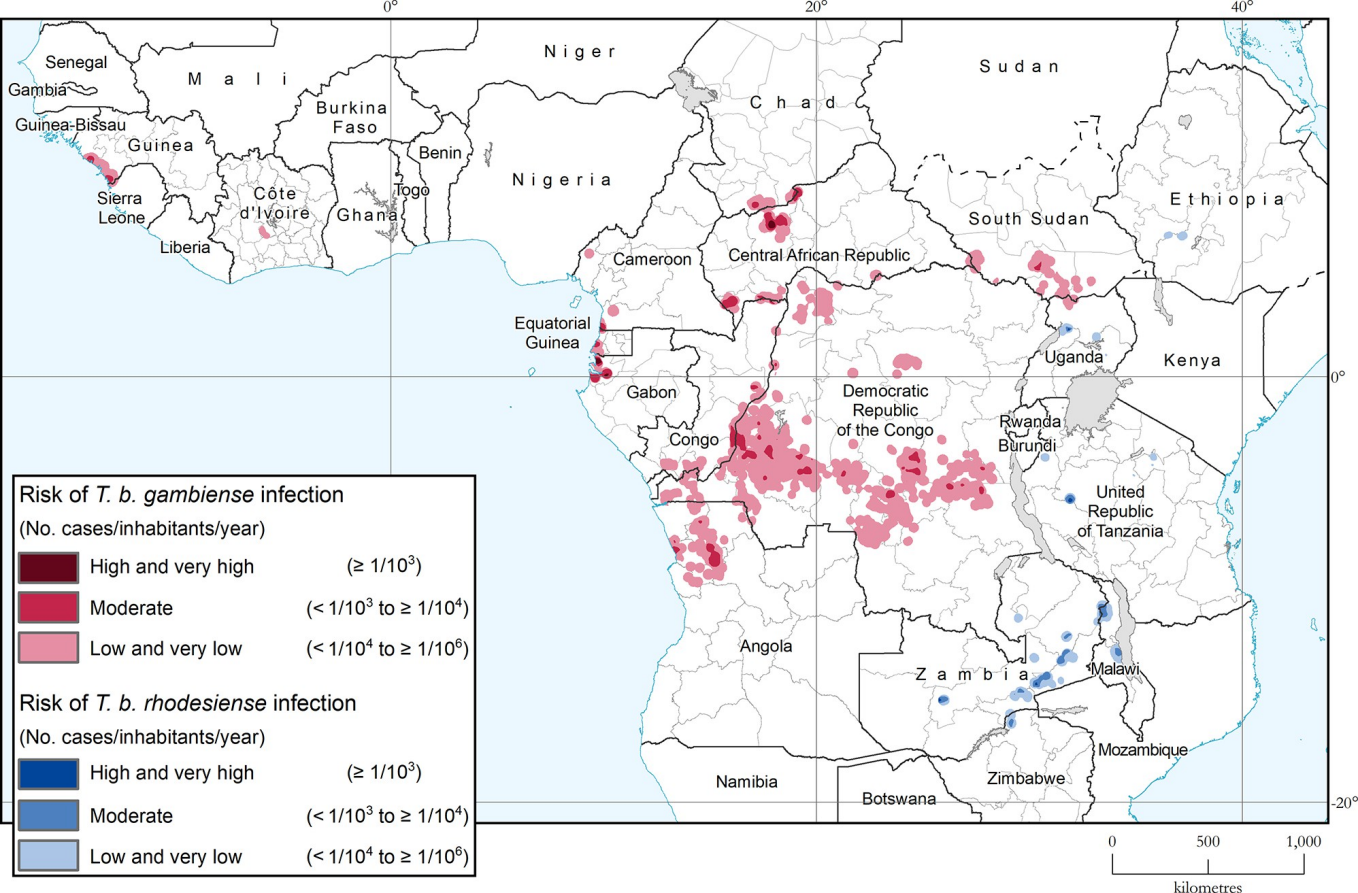
Areas at risk of HAT infection, 2018–2022. The base layers used in this map are the FAO Global Administrative Unit Layers (GAUL) https://data.apps.fao.org/map/catalog/srv/eng/catalog.search#/metadata/9c35ba10-5649-41c8-bdfc-eb78e9e65654, FAO Inland water bodies in Africa https://data.apps.fao.org/map/catalog/srv/eng/catalog.search;jsessionid=B7AF7A215B16770A1A67C65D97FF21CA?node=srv#/metadata/bd8def30-88fd-11da-a88f-000d939bc5d8 and subnational divisions from The Humanitarian Data Exchange (OCHA) https://data.humdata.org/dataset/?vocab_Topics=administrative+boundaries-divisions.

The areas reporting ≥ 1 HAT case/10 000 inhabitants/year for the period 2018–2022 cover a surface of 73,134 km^2^, with only 3013 km^2^ at very high or high risk (Fig 3). This represents a reduction of 90% from the baseline figure for 2000–2004 and, therefore, the targets set for the elimination of HAT as a public health problem at the global level can be considered fully reached.

The area at moderate or higher risk is 58 221 km^2^ for gambiense HAT (91% reduction from the 2000–2004 baseline) and 14 913 km^2^ for rhodesiense HAT (46% reduction). These areas are distributed mainly in the Democratic Republic of the Congo (30%) but also in Central African Republic (17.5%), Zambia (12%), Angola (9.5%), Gabon, (7.9%), Congo (5.2%), Malawi (4.1%), Guinea (2.9%), United Republic of Tanzania (2.4%), Chad (2.2%), Cameroon (1.6%), Equatorial Guinea (1.5%), South Sudan (1.3%), Zimbabwe (1.0%) and Uganda (0.8%) (S1 Table, S2 Fig).

The few remaining areas at high or very high risk are located in Central African Republic (1188 km^2^ in Kambakota-Batangafo area in the Ouham focus) and Gabon (1028 km^2^ in Noya and Komo Kango in Estuaire focus) for gambiense HAT, and in Uganda (113 km^2^ in the Murchison Falls National Park), western Tanzania (365 km^2^ in the Ugalla River Game Reserve), and Zambia (317 km^2^ in small areas inside the Kafue National Park and the West Petauke Game Management Area) for rhodesiense HAT (S1 Table, S2 Fig).

The total population at risk of HAT in 2018–2022 is estimated at 41.5 million, with only 4% (1.5 million people) at moderate or higher risk. Out of them 38.9 million (94%) are at risk of gambiense HAT and 2.6 million (6%) of the rhodesiense form. Along with the reduction of the area at risk, the population at risk of HAT has progressively decreased: in the period 2000–2004, there were 55.9 million people living in areas at risk, with 5.8 million living in areas at high or very high risk (S2 Table).

### Control and surveillance activities: coverage of the at-risk population

#### Active surveillance

**Fig 4:**
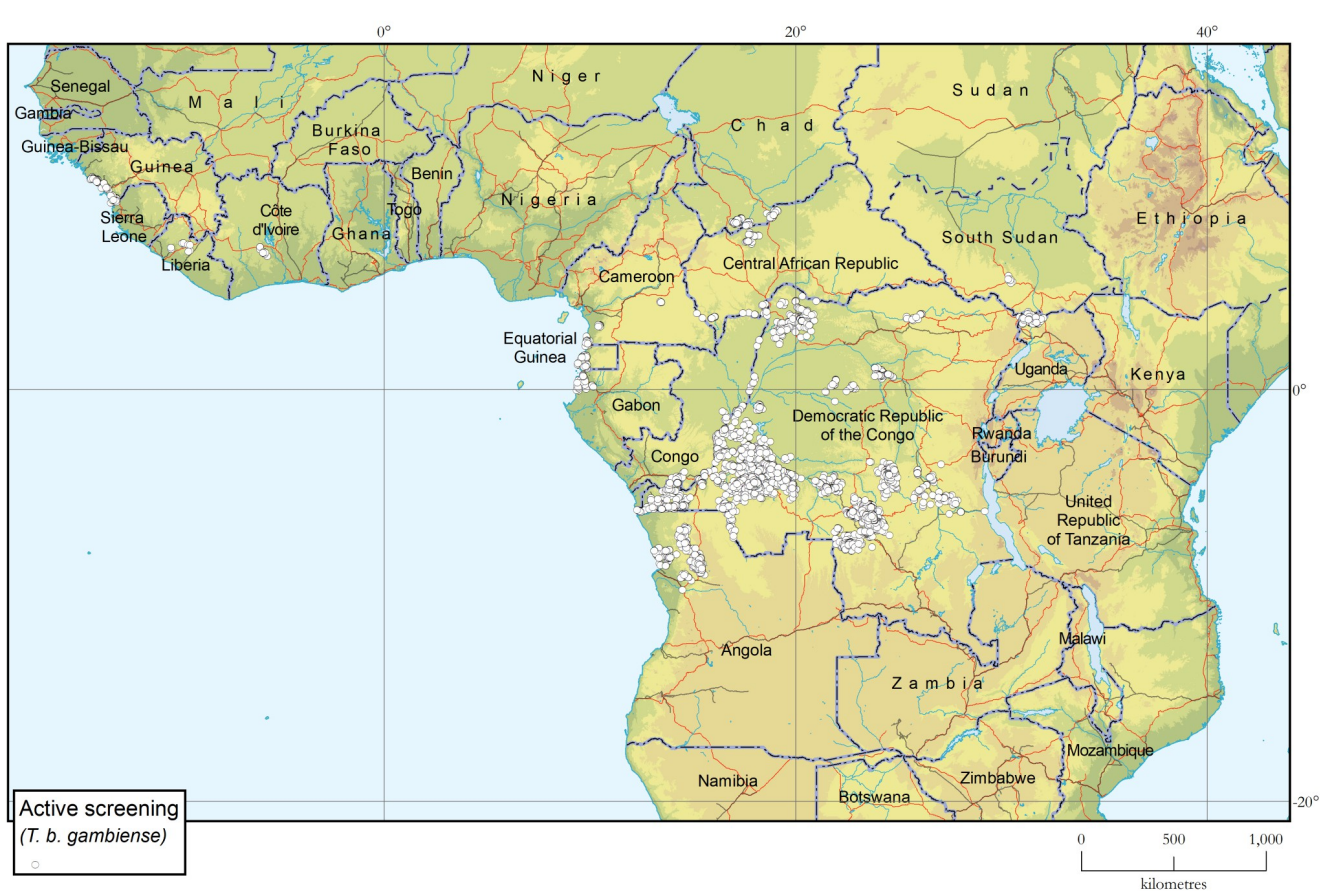
Locations where active screening for gambiense HAT performed, 2018–2022. The base layers used in this map are the FAO Global Administrative Unit Layers (GAUL) https://data.apps.fao.org/map/catalog/srv/eng/catalog.search#/metadata/9c35ba10-5649-41c8-bdfc-eb78e9e65654, Shuttle Radar Topography Mission (SRTM) https://doi.org/10.5066/F7F76B1X, FAO Inland water bodies in Africa https://data.apps.fao.org/map/catalog/srv/eng/catalog.search;jsessionid=B7AF7A215B16770A1A67C65D97FF21CA?node=srv#/metadata/bd8def30-88fd-11da-a88f-000d939bc5d8, FAO Rivers of Africa https://data.apps.fao.org/map/catalog/srv/eng/catalog.search;jsessionid=B7AF7A215B16770A1A67C65D97FF21CA?node=srv#/metadata/b891ca64-4cd4-4efd-a7ca-b386e98d52e8 and Vector Map Level 0 (VMap0) https://gis-lab.info/qa/vmap0-eng.html.

**Fig 5:**
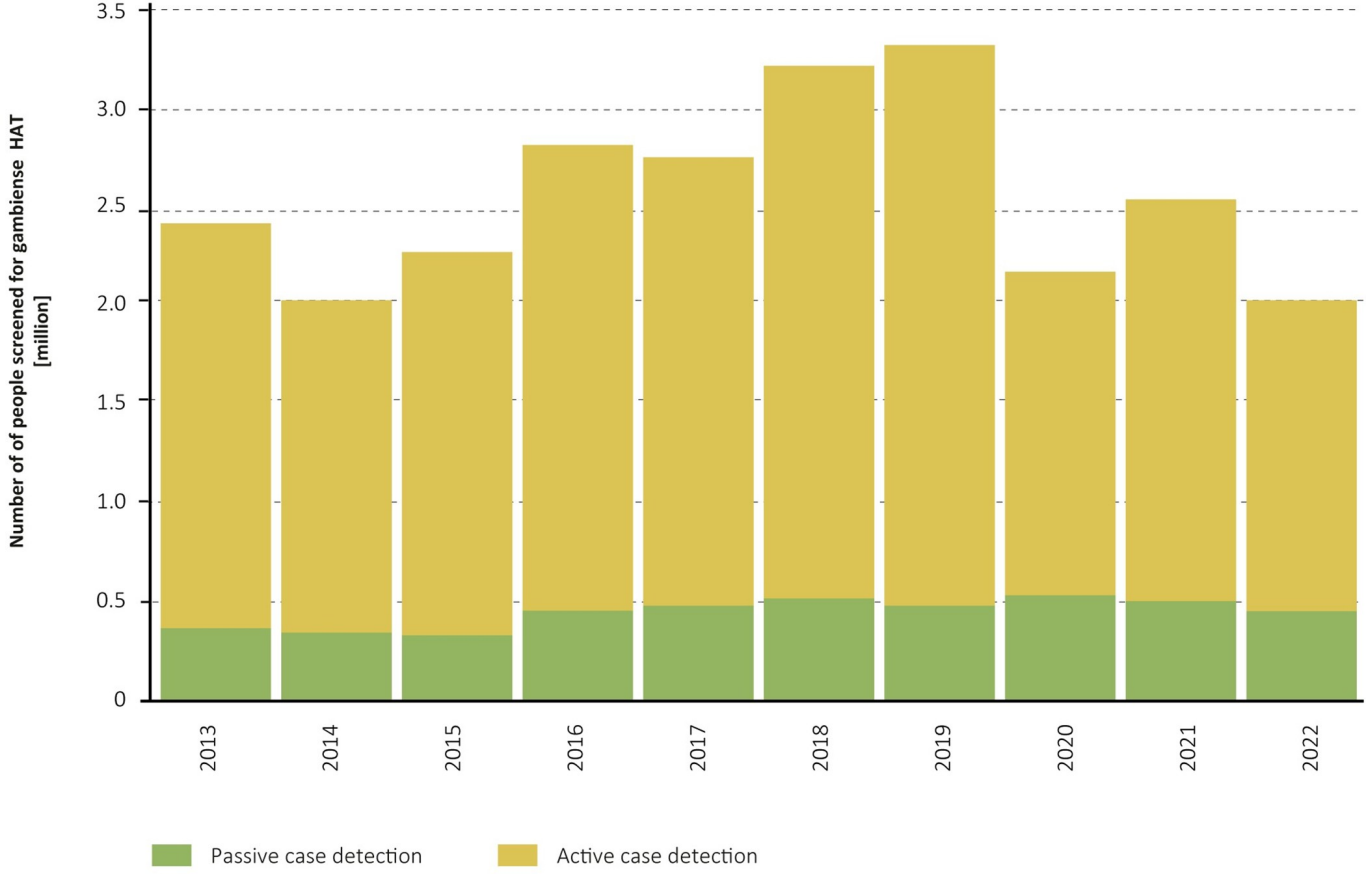
Number of people screened for gambiense HAT, 2013–2022.

A total of 3 599 039 people were screened for HAT in 2021–2022 (i.e. 2 033 969 in 2021 and 1 565 070 in 2022). This is the lowest number of people actively screened in the past 10 years, with a 22% reduction from 2019–2020. A 92% of the population actively screened in the biennium was in the Democratic Republic of the Congo. The intensity of active screening has been decreasing in the past years because of the strategic reduction of mobile teams but also because of problems in financial management (Figs 4 and 5).

#### Passive surveillance

According to the reports provided by the NSSCPs, a total of 1521 fixed health facilities with capacity for HAT diagnosis were inventoried in 2022. As these figures were obtained from country reports and not through a specific survey, direct comparisons with the results of previous surveys cannot be made.

For gambiense HAT, 1294 fixed health facilities offering diagnosis were reported in 2022, with 796 in the Democratic Republic of the Congo. Facilities for HAT diagnosis were set up in three new countries: Liberia (3), Ethiopia (9) and Senegal (15).

In 2021–2022 a total of 900 407 people were passively screened for gambiense HAT with serological tests, thus complementing the active screening (Fig 5), of whom 93% were tested in the Democratic Republic of the Congo. These figures are very similar to those of previous reporting periods, with 1 004 785 passively screened in 2019–2020 and 986 920 in 2017–2018.

For rhodesiense HAT, diagnosis was reported to be available from a total of 221 facilities, including Kenya, Malawi, Rwanda, Uganda, United Republic of Tanzania, Zambia, Zimbabwe and, as of 2022, Ethiopia.

It is important to highlight that the HAT control protocol of some countries classifies individuals with high titration of serological tests (CATT), within foci of active transmission, as probable cases even if parasitological tests are negative. They receive treatment and are reported as cases unconfirmed by parasitology (no trypanosomes seen). This constitutes a more aggressive HAT elimination strategy, on one hand, and a compensation for a weak microscopy capacity in some areas. Considering both active and passive surveillance, in 2021–2022 91.8% of the gambiense HAT cases were cases confirmed by parasitology. The highest rates of parasitologically unconfirmed cases were in Chad (72.7%), Congo (64.3%), South Sudan (47.5%), Cameroon (38.9%) and Central African Republic (36.1%).

### Case management

There were 682 health facilities reported as centres for treatment of HAT in 2022 in 20 countries. As for diagnosis facilities, the figures on treatment facilities were obtained from country reports and not through a survey as on previous occasions, and therefore direct comparisons are not warranted.

A total of 652 facilities can provide treatment for gambiense HAT in 13 countries (Angola, Burkina Faso, Cameroon, Central African Republic, Chad, Congo, Côte d’Ivoire, Democratic Republic of the Congo, Equatorial Guinea, Gabon, Guinea, South Sudan and Uganda). For rhodesiense HAT, treatment can be obtained in 30 facilities in seven countries (Ethiopia, Kenya, Malawi, Uganda, United Republic of Tanzania, Zambia and Zimbabwe).

No specific health facility with capacity to treat or indeed diagnose HAT has been reported from Botswana, Burundi, Eswatini, the Gambia, Guinea-Bissau, Mozambique, Namibia, Niger and Sierra Leone.

For gambiense HAT, 1473 cases were reported as treated (95.3% of detected cases), of which 641 (43.52%) cases received fexinidazole, 527 (35.78%), nifurtimox–eflornithine combination therapy and 305 (20.71%) pentamidine. For rhodesiense HAT, 97 cases were reported as treated, including 6 relapses, of whom 60 (61.86%) received melarsoprol, 26 (26.8%) suramin and 11 (11.34%) fexinidazole (in clinical trials).

### Vector control

Tsetse control has been historically a key component in the control of animal trypanosomiasis, while it has been used much less to control sleeping sickness, especially of the gambiense form. However, in recent years, more vector control activities were implemented by several NSSCPs and their partners with a view to contributing to gambiense HAT control and elimination [33]. This was the case in Angola, Cameroon [46], Chad [47–49], Côte d’Ivoire [49–51], Democratic Republic of the Congo [52–54], Guinea [49] and Uganda [41,45,55].

For rhodesiense HAT endemic countries, vector control activities implemented in coordination with the NSSCP were carried out in Malawi. In several other countries there exist multisectoral bodies, often established within a “One Health” framework, which include the Ministries of health and the NSSCP and the Ministries of Agriculture and Livestock. These bodies promote or implement vector control activities, mainly targeting animal trypanosomiasis or else to control the tsetse nuisance in national parks or other protected areas, but in so doing, they also contribute to HAT control. This is the case in Ethiopia [45], Kenya [56–58], Rwanda [59–61], United Republic of Tanzania [62], Uganda [63–65], Zambia [66–68] and Zimbabwe [69].

### Number of countries validated for elimination of HAT as a public health problem

In 2021 the elimination of HAT as a public health problem was validated in Benin and Uganda for gambiense HAT and in Rwanda for rhodesiense HAT [70]. In 2022, validation was also endorsed for Equatorial Guinea and Ghana [71]. These countries follow Togo and Côte d’Ivoire [72], which were both validated in 2020. In 2022, one additional country (Chad) submitted its dossier to WHO for validation, while Burkina Faso, Kenya and Guinea were in the process of preparing dossiers. A summary of the status of country validations is shown in Table 3.

**Table 3:** Status of HAT elimination as a public health problem at the country level in 2022.

	Elimination as a public health problem still not reached	Elimination as a public health problem reached but surveillance insufficient	Elimination as a public health problem reached and dossier ready to submit for validation	Elimination as a public health problem reached and dossier submitted for validation	Elimination as a public health problem validated and post-validation surveillance implemented
**Gambiense HAT**	Angola, Central African Republic, Congo, Democratic Republic of the Congo	Gambia, Guinea Bissau, Liberia, Mali, Niger, Nigeria, Senegal, Sierra Leone, South Sudan	Burkina Faso, Cameroon, Gabon, Guinea	Chad	Benin, Côte d’Ivoire, Equatorial Guinea, Ghana, Togo, Uganda
**Rhodesiense HAT**	Malawi	Botswana, Burundi, Eswatini, Ethiopia, Kenya, Mozambique, Namibia, Uganda, United Republic of Tanzania, Zambia, Zimbabwe	Kenya		Rwanda

The validation of elimination of HAT as a public health problem at country level requires the establishment of post-elimination surveillance systems. In Togo, after the evaluation of reported data by the NSSCP and WHO, post-elimination activities were considered weak, hence efforts to reinforce them were put in place. Interestingly, this surveillance was able to detect a notable increase in the number of HAT cases in the focus of Mbini in Equatorial Guinea, thus triggering the deployment of targeted control measures.

### The 2021–2030 WHO road map: verification of elimination of transmission at country level

The elimination of transmission of gambiense HAT is a goal at the country level. To reach it, countries have to report zero cases of human infection with *T*. *b*. *gambiense* for a period of at least 5 consecutive years, with evidence of an adequate HAT surveillance system. Countries claiming the elimination of gambiense HAT transmission should submit a dossier to WHO for its verification; the template for the dossier was defined by a technical advisory group of WHO. The details of the template and of the related verification process were recently published [36], and it is expected that countries will start to engage in formal verification. In coming years, the number of countries verified in this way for elimination of transmission will be a key indicator of progress towards the target set for 2030.

## Discussion

The epidemiological patterns of HAT in the biennium 2021–2022 show that recent advances in disease control are being sustained, with fewer than 1000 cases declared in both 2021 and 2022, within a continuous decreasing trend against a baseline of more than 25,000 cases per year in the biennium 2000–2001, when systematic and comprehensive data collection started. The goal of the global elimination of HAT as a public health problem, set in the first road map in 2012 by WHO and endemic countries, was reached. At the national level, the elimination of HAT as a public health problem has been validated in seven countries (Côte d’Ivoire, Togo, Benin, Uganda, Equatorial Guinea and Ghana for gambiense HAT and Rwanda for rhodesiense HAT). In line with this trend, the population living in areas at risk of HAT is continuing to decrease, being currently estimated at 41.5 million people, with the majority (40.0 million) at low or very low risk.

It is crucial to highlight that these achievements do not mean that control and surveillance activities can be stopped. Rather, activities should be maintained and, whenever possible, enhanced, to ensure that advances are sustained, that any possible re-emergence or re-introduction of the disease is detected, and that further progress towards the elimination of transmission is made. Monitoring of field activities is essential, because it triggers an alert when a reduction in intensity is observed (e.g. Togo) or when a recrudescence of the disease occurs (e.g. the Mbini focus in Equatorial Guinea). At the same time, the validation of the elimination of HAT as a public health problem must be promoted and supported in other countries.

Even if advances in the control and surveillance of HAT are widespread, the situation in a few areas remains a cause for concern. A more stable security situation allowed control and surveillance in Central African Republic and South Sudan to be improved, but further progress is still needed. Improvements in HAT activities are also clearly reported in Angola and Gabon, but further efforts are still needed. The Democratic Republic of the Congo showed notable advances but the magnitude of the HAT problem in the country, and its vast territorial expanse, requires substantial efforts to sustain and enhance the achievements. Important shortcomings in HAT surveillance and control persist in Congo and Nigeria.

The achievement of HAT elimination as a public health problem at the global level obviously means that the disease is no longer a public health problem in Africa as a whole. However, a negative consequence of this success is that health staff knowledge of the disease is decreasing, as experienced staff progressively retire and new staff have fewer opportunities to encounter HAT. An increasingly rare disease is less likely to be considered in differential diagnoses. Furthermore, many affected communities no longer perceive HAT as a major health concern and competing health problems diminish their involvement in HAT control activities. Finally, HAT is no longer a priority for political and health authorities in charge of decision-making and investments in public health.

Despite these constraints, there is a general commitment by NSSCPs and partners to reach the elimination of gambiense HAT transmission by 2030, represented by the tantalizing prospect of zero cases reported. In the current context of very low prevalence, trying to reach this target is well justified but poses enormous challenges.

New control tools in the pipeline could be very helpful in fine-tuning and enhancing strategies towards reaching this target. In particular, given the impossibility of maintaining costly vertical programmes, there is a strong need to integrate HAT control and surveillance into the primary health system, which in gambiense HAT endemic areas is usually very weak and poorly endowed. To succeed with such integration, simpler diagnostic and therapeutic tools that can be used by non-specialized health staff are urgently needed [73–76]. At the same time, together with the integration in peripheral health services, and notwithstanding the small number of cases, it is important to ensure the quality of surveillance. Reliable remote diagnostic tests and reference centres able to perform them are also needed to identify infected individuals [77–81]. Finally, simple and reliable methods to refer these samples without a cold chain are needed.

Screening for cases needs to be very carefully targeted, covering those areas where cases are more likely to be found or, as reactive screening, those areas where sporadic cases are still detected. In areas where epidemiological knowledge is poor, active screening can be implemented in the form of population surveys, which are helpful in providing a general appraisal of the situation; in these settings high throughput tests would be advantageous [82,83].

Strategies could be improved to tackle the possible cryptic reservoirs. In the case of asymptomatic infected people, their detection and management could be improved (e.g. if safer and simpler medicines were available, treatment of gambiense HAT serological suspects without parasitological confirmation could be considered in some settings). In the management of a possible animal reservoir, treatment of animals, protecting animals with impregnated fences or restricted insecticide applications could be considered in some areas.

Targeted vector control activities in priority areas with affordable methods, adapted to local conditions and associated to other HAT control methods, will also contribute to achieving the elimination of the disease. However, the long-term sustainability of these interventions remains a challenge, and careful targeting will probably be needed also in terms of the timing of vector control.

In a context of the disease no longer being a public health problem, national authorities from endemic countries should now reaffirm their commitment to achieving elimination by assuming stronger ownership of this target and providing the support and at least the minimal resources needed.

Bearing in mind that currently the disease is no longer considered a public health problem in many endemic areas, it is still important to keep the collaboration of community actors in control and surveillance activities, taking into account key sociocultural aspects to ensure the required engagement and participation, and adapting interventions to keep these actors in the front line of the elimination process.

A risk inherent in having achieved elimination as a public health problem is “donor fatigue”. It is not inconceivable that after many years of investment and considering that a first stated goal has been achieved, donor support may decrease. However, it is important to keep a long-term commitment of donors, building on the results obtained and communicating future objectives effectively. Since 2001, the continuous donation of all the medicines needed to ensure the treatment of all HAT detected cases has been crucial for the elimination of HAT as a public health problem, and it should be maintained now that the number of people requiring treatment is very small. Extending this donation to diagnostic tools would further improve efforts to sustain the downward pressure on disease.

All interventions and strategies involving different partners require coordination of stakeholders with different agendas, in order to avoid overlaps or disruptions, and to maximize synergies. WHO has effectively provided coordination and guidance in the past, and it is committed to continuing their provision in the future.

Past experiences and the ever-present possibility of unexpected events (e.g. Ebola virus disease or COVID-19 epidemics, civil strife) require building resilience with a view to minimizing the potential negative impacts of these events.

As regards rhodesiense HAT, despite its low case-number, it should not be neglected, and efforts to develop better tools for diagnosis [84,85] and treatment, adapted to the current context, should be sustained. Currently diagnostic capacity for rhodesiense HAT is weak in most endemic countries; this, coupled with the acute progression of the disease, may lead to a relatively high level of under-detection. The control of rhodesiense HAT could be enhanced substantially by integrating interventions for progressive animal trypanosomiasis control [86] and for safer tourism. The experience of unexpected outbreaks such as that occurring by Ethiopia in 2022 should be considered in designing strategies to reinforce capacities for detection and quick reaction.

## Conclusions

HAT elimination as a public health problem was reached at global level, with seven countries already validated. This achievement was made possible by the work of NSSCPs, supported by different public and private partners, and coordinated by WHO. WHO continues to support the process of HAT elimination as a public health problem in other endemic countries. Now, the road map target for eliminating the transmission of gambiense HAT is being pursued, with the challenging goal of reaching zero cases in 2030.

Despite the imperfect tools currently available and the limited resources invested, gambiense HAT is one of the diseases for which advances towards elimination have been the most striking in past years.

Along with the focus on elimination of transmission of gambiense HAT, it is important not to neglect rhodesiense HAT, which is targeted for elimination as a public health problem in the WHO road map for NTDs 2021–2030. The current tools for the diagnosis and treatment of rhodesiense HAT are poor, and advances are needed to improve its surveillance (simple and easy to perform tests) and case management (safer and easier treatments). Control of this zoonotic form of HAT will be possible only under a One Health approach focusing on the animal reservoirs and the vectors. At the same time, it is important to maintain capacity for quick reactivity to rapidly control possible outbreaks.

The ambitious target set for 2030 should motivate donors and stakeholders to maintain their engagement and support. There is a commitment by pharmaceutical companies to ensure access to medicines (both existing ones and new ones in the pipeline). Nevertheless, the challenges are many, in particular the improvement and the availability of diagnostics. Above all, it is crucial to keep the active involvement and coordination of all partners, starting with national control programmes and health authorities in endemic countries, research institutions, implementing partners and donors. With this sustained commitment and coordination, further progress towards the elimination goals will be possible.

## Disclaimers

The boundaries and names shown and the designations used on the maps presented in this paper do not imply the expression of any opinion whatsoever on the part of WHO and FAO concerning the legal status of any country, territory, city or area or of its authorities, or concerning the delimitation of its frontiers or boundaries.

The authors alone are responsible for the views expressed in this article and they do not necessarily represent the views, decisions or policies of the institutions with which they are affiliated.

## Supporting information

S1 Table Area at risk of gambiense and rhodesiense HAT. Period 2018–2022 (by country).
(DOCX)

S2 Table Population at risk of gambiense and rhodesiense HAT. Period 2018–2022 (by country).
(DOCX)

S1 Fig Geographical distribution of human African trypanosomiasis, subregional maps (PDF). Period 2021–2022. The base layers used in the maps are the FAO Global Administrative Unit Layers (GAUL), Global Administrative Areas, Shuttle Radar Topography Mission (SRTM), FAO Inland water bodies in Africa, FAO Rivers of Africa and Vector Map Level 0 (VMap0).
(PDF)

S2 Fig Areas at risk of HAT infection, subregional maps. Period 2016–2020 (PDF). The base layers used in the maps are the FAO Global Administrative Unit Layers (GAUL), Global Administrative Areas and FAO Inland water bodies in Africa.
(PDF)
