# Supplementary material for: The elimination of human African trypanosomiasis: Monitoring progress towards the 2021–2030 WHO road map targets
Source: PLoS Negl Trop Dis. 2024 Apr 16;18(4):e0012111. doi: 10.1371/journal.pntd.0012111 (PMC11073784; doi:10.1371/journal.pntd.0012111)
Supplement: S1 Table — Period 2018–2022 (by country). (DOCX) [file pntd.0012111.s001.docx]

# Area at risk of gambiense and rhodesiense HAT

Table 1 Areas at risk of *T. b. gambiense* infection (km^2^). Period 2018–2022.

| **Country** | **Total country area*** | **Area at risk**  **2018-2022** | | | | |
| --- | --- | --- | --- | --- | --- | --- |
|  |  | **Very High**  **and High** | **Moderate** | **Low and**  **Very Low** | **Total**  **at risk** | **% of total**  **country**  **area** |
| Angola | 1,253,770 | 0 | 6,928 | 59,093 | 66,021 | 5.3 |
| Burkina Faso | 274,470 | 0 | 0 | 0 | 0 | 0 |
| Cameroon | 466,396 | 0 | 1,154 | 6,375 | 7,529 | 1.6 |
| Central African Republic | 624,398 | 1,188 | 11,590 | 28,246 | 41,025 | 6.6 |
| Chad | 1,272,490 | 0 | 1,613 | 9,560 | 11,172 | 0.9 |
| Congo | 338,522 | 0 | 3,792 | 28,327 | 32,120 | 9.5 |
| Côte d'Ivoire | 321,363 | 0 | 0 | 2,299 | 2,299 | 0.7 |
| Democratic Republic of the Congo | 2,304,080 | 0 | 21,972 | 406,935 | 428,907 | 18.6 |
| Equatorial Guinea | 27,019 | 0 | 1,131 | 3,538 | 4,669 | 17.3 |
| Gabon | 265,978 | 1,028 | 4,751 | 3,770 | 9,549 | 3.6 |
| Guinea | 246,094 | 0 | 2,108 | 8,524 | 10,632 | 4.3 |
| Sierra Leone | 72,777 | 0 | 0 | 1,194 | 1,194 | 1.6 |
| South Sudan | 633,356 | 0 | 964 | 36,358 | 37,322 | 5.9 |
| Uganda | 205,540 | 0 | 0 | 3,754 | 3,754 | 1.8 |
| Other Endemic Countries** | 4,097,446 | 0 | 0 | 0 | 0 | 0 |
| Total | 12,403,699 | 2,217 | 56,004 | 597,972 | 656,193 | 5.3 |

* Land area. The area of surface water bodies as depicted in the Shuttle Radar Topography Mission—River-Surface Water Bodies dataset is not included.

** Countries at marginal risk: Benin, Gambia, Ghana, Guinea-Bissau, Liberia, Mali, Niger, Nigeria, Senegal and Togo.

Table 2 Areas at risk of *T. b. rhodesiense* infection (km^2^). Period 2018–2022.

| **Country** | **Total country area*** | **Area at risk**  **2018-2022** | | | | |
| --- | --- | --- | --- | --- | --- | --- |
|  |  | **Very High**  **and High** | **Moderate** | **Low and**  **Very Low** | **Total**  **at risk** | **% of total**  **country**  **area** |
| Ethiopia | 1,126,970 | 0 | 0 | 3,430 | 3,430 | 0.3 |
| Malawi | 94,758 | 0 | 3,032 | 6,847 | 9,879 | 10.4 |
| United Republic of Tanzania | 886,278 | 366 | 1,402 | 3,754 | 5,521 | 0.6 |
| Uganda | 205,540 | 114 | 478 | 4,605 | 5,197 | 2.5 |
| Zambia | 742,479 | 317 | 8,448 | 26,958 | 35,723 | 4.8 |
| Zimbabwe | 388,414 | 0 | 756 | 2,700 | 3,456 | 0.9 |
| Other Endemic Countries** | 2,823,315 | 0 | 0 | 0 | 0 | 0 |
| Total | 6,267,754 | 796 | 14,117 | 48,293 | 63,206 | 1.0 |

* Land area. The area of surface water bodies as depicted in the Shuttle Radar Topography Mission—River-Surface Water Bodies dataset is not included.

** Countries at marginal risk: Botswana, Burundi, Eswatini, Kenya, Mozambique, Namibia and Rwanda.
