# Supplementary material for: The elimination of human African trypanosomiasis: Monitoring progress towards the 2021–2030 WHO road map targets
Source: PLoS Negl Trop Dis. 2024 Apr 16;18(4):e0012111. doi: 10.1371/journal.pntd.0012111 (PMC11073784; doi:10.1371/journal.pntd.0012111)
Supplement: S2 Table — Period 2018–2022 (by country). (DOCX) [file pntd.0012111.s002.docx]

# Population at risk of gambiense and rhodesiense HAT

Table 1 Population at risk of *T. b. gambiense* infection (no. persons × 10^3^). Period 2018–2020.

| **Country** | **Total country population**  **2022*** | **Population at risk**  **2018-2022** | | | | |
| --- | --- | --- | --- | --- | --- | --- |
|  |  | **Very High**  **and High** | **Moderate** | **Low and**  **Very Low** | **Total**  **at risk** | **% of total**  **country**  **population** |
| Angola | 34,689 | 0 | 111 | 2,328 | 2,439 | 7.0 |
| Burkina Faso | 21,948 | 0 | 0 | 0 | 0 | 0 |
| Cameroon | 29,337 | 0 | 44 | 158 | 202 | 0.7 |
| Central African Republic | 5,434 | 5 | 128 | 455 | 587 | 10.8 |
| Chad | 17,996 | 0 | 27 | 483 | 509 | 2.8 |
| Congo | 5,526 | 0 | 17 | 711 | 728 | 13.2 |
| Côte d'Ivoire | 28,571 | 0 | 0 | 395 | 395 | 1.4 |
| Democratic Republic of the Congo | 108,384 | 0 | 758 | 28,747 | 29,505 | 27.2 |
| Equatorial Guinea | 1,670 | 0 | 33 | 36 | 69 | 4.1 |
| Gabon | 2,305 | 4 | 14 | 54 | 72 | 3.1 |
| Guinea | 12,915 | 0 | 95 | 2,390 | 2,485 | 19.2 |
| Sierra Leone | 8,560 | 0 | 0 | 247 | 247 | 2.9 |
| South Sudan | 11,575 | 0 | 11 | 1,201 | 1,212 | 10.5 |
| Uganda | 46,289 | 0 | 0 | 437 | 437 | 0.9 |
| Other Endemic Countries** | 351,637 | 0 | 0 | 0 | 0 | 0 |
| Total | 686,835 | 9 | 1,239 | 37,641 | 38,889 | 5.7 |

* As per Landscan

** Countries at marginal risk: Benin, Gambia, Ghana, Guinea-Bissau, Liberia, Mali, Niger, Nigeria, Senegal and Togo.

Table 2 Population at risk of *T. b. rhodesiense* infection (no. persons × 10^3^). Period 2018–2022.

| **Country** | **Total country population**  **2022*** | **Population at risk**  **2018-2022** | | | | |
| --- | --- | --- | --- | --- | --- | --- |
|  |  | **Very High**  **and High** | **Moderate** | **Low and**  **Very Low** | **Total**  **at risk** | **% of total**  **country**  **population** |
| Ethiopia | 113,698 | 0 | 0 | 255 | 255 | 0.2 |
| Malawi | 20,785 | 0 | 247 | 750 | 998 | 4.8 |
| United Republic of Tanzania | 63,604 | 0 | 1 x 10^-1^ | 238 | 238 | 0.4 |
| Uganda | 46,289 | 0 | 2 x 10^-2^ | 538 | 538 | 1.2 |
| Zambia | 19,647 | 0 | 15 | 554 | 569 | 2.9 |
| Zimbabwe | 15,099 | 0 | 1 x 10^-1^ | 7 x 10^-1^ | 8 x 10^-1^ | 5.3 x 10^-3^ |
| Other Endemic Countries** | 119,338 | 0 | 0 | 0 | 0 | 0 |
| Total | 398,461 | 0 | 263 | 2,336 | 2,599 | 0.7 |

* As per Landscan

** Countries at marginal risk: Botswana, Burundi, Eswatini, Kenya, Mozambique, Namibia and Rwanda.
