# Supplementary material for: The elimination of human African trypanosomiasis: Monitoring progress towards the 2021–2030 WHO road map targets
Source: PLoS Negl Trop Dis. 2024 Apr 16;18(4):e0012111. doi: 10.1371/journal.pntd.0012111 (PMC11073784; doi:10.1371/journal.pntd.0012111)
Supplement: S1 Fig — Period 2021–2022. The base layers used in the maps are the FAO Global Administrative Unit Layers (GAUL), Global Administrative Areas, Shuttle Radar Topography Mission (SRTM), FAO Inland water bodies in Africa, FAO Rivers of Africa and Vector Map Level 0 (VMap0). (PDF) [file pntd.0012111.s003.pdf]

Gambiense HAT cases in Western Africa. Period 2021–2022.

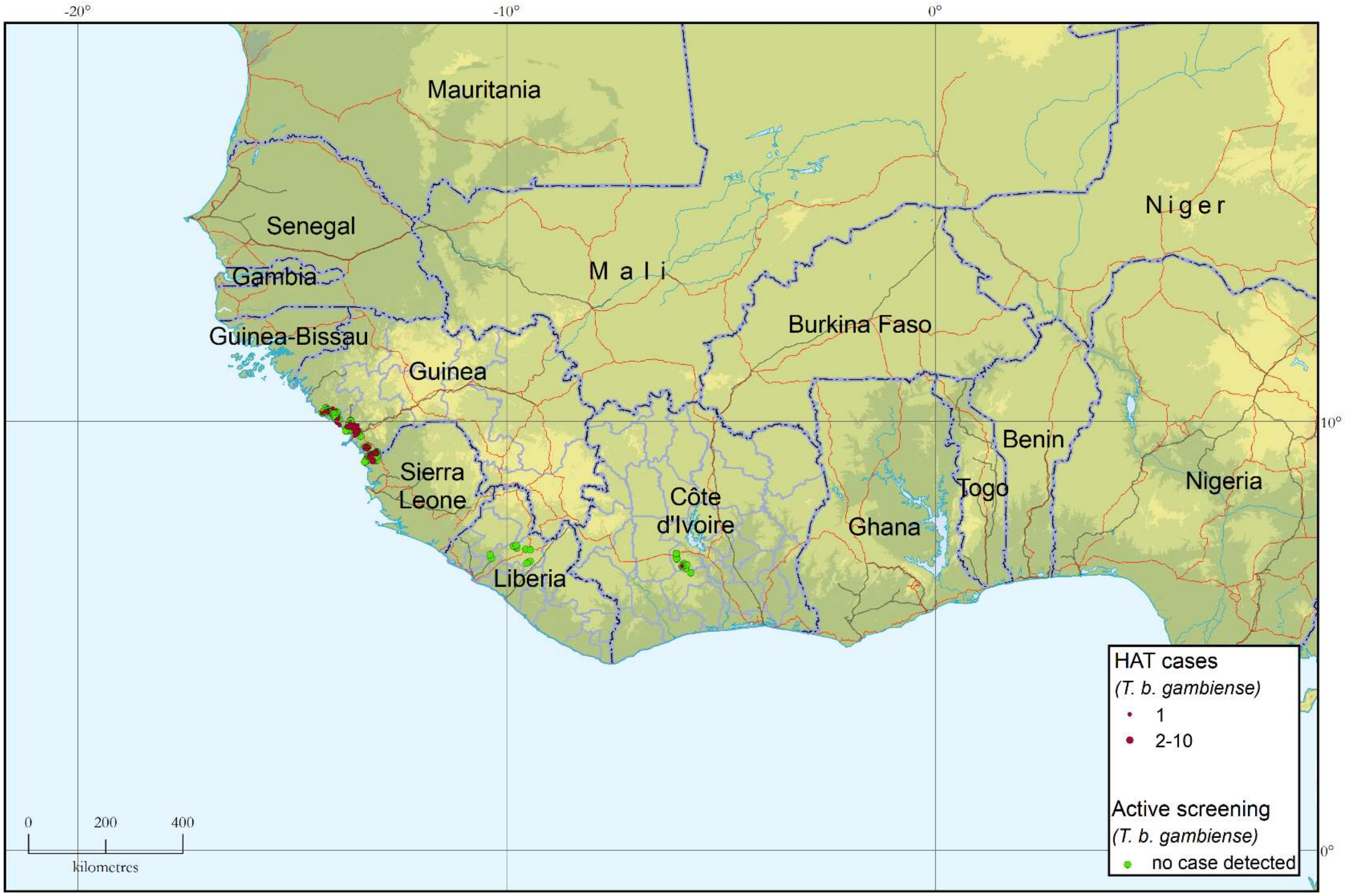

The base layers used in this map are the FAO Global Administrative Unit Layers (GAUL) https://data.apps.fao.org/map/catalog/srv/eng/catalog.search#/metadata/9c35ba10-5649-41c8-bdfc-eb78e9e65654, Shuttle Radar Topography Mission (SRTM) https://doi.org/10.5066/F7F76B1X, FAO Inland water bodies in Africa https://data.apps.fao.org/map/catalog/srv/eng/catalog.search;jsessionid=B7AF7A215B16770A1A67C65D97FF21CA?node=srv#/metadata/bd8def30-88fd-11da-a88f-000d939bc5d8, FAO Rivers of Africa https://data.apps.fao.org/map/catalog/srv/eng/catalog.search;jsessionid=B7AF7A215B16770A1A67C65D97FF21CA?node=srv#/metadata/b891ca64-4cd4-4efd-a7ca-b386e98d52e8, Vector Map Level 0 (VMap0) https://gis-lab.info/qa/vmap0-eng.html and subnational divisions from The Humanitarian Data Exchange (OCHA) https://data.humdata.org/dataset/?vocab_Topics=administrative+boundaries-divisions.

Gambiense HAT cases in Central Africa. Period 2021–2022.

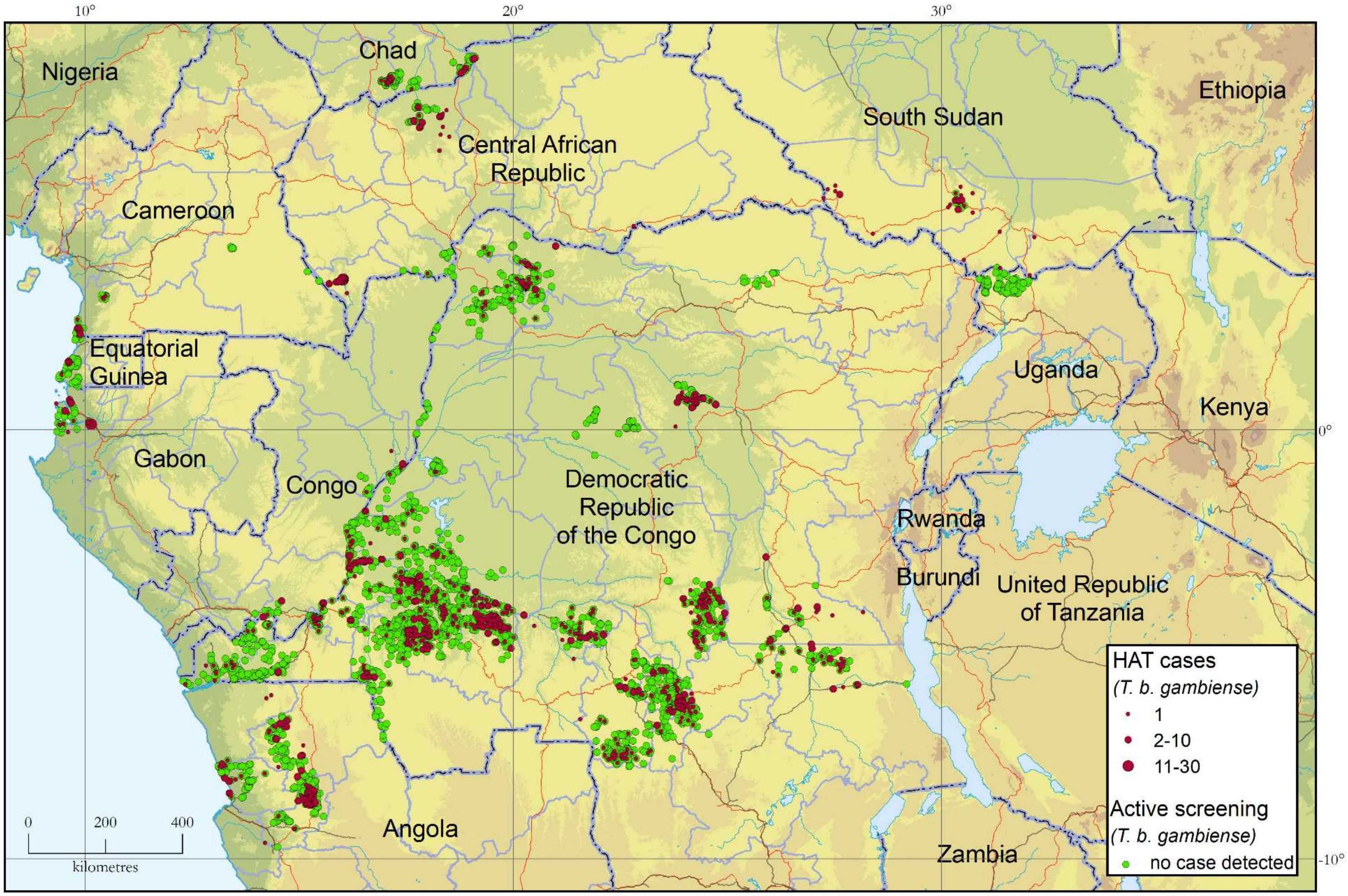

The base layers used in this map are the FAO Global Administrative Unit Layers (GAUL) https://data.apps.fao.org/map/catalog/srv/eng/catalog.search#/metadata/9c35ba10-5649-41c8-bdfc-eb78e9e65654, Shuttle Radar Topography Mission (SRTM) https://doi.org/10.5066/F7F76B1X, FAO Inland water bodies in Africa https://data.apps.fao.org/map/catalog/srv/eng/catalog.search;jsessionid=B7AF7A215B16770A1A67C65D97FF21CA?node=srv#/metadata/bd8def30-88fd-11da-a88f-000d939bc5d8, FAO Rivers of Africa https://data.apps.fao.org/map/catalog/srv/eng/catalog.search;jsessionid=B7AF7A215B16770A1A67C65D97FF21CA?node=srv#/metadata/b891ca64-4cd4-4efd-a7ca-b386e98d52e8, Vector Map Level 0 (VMap0) https://gis-lab.info/qa/vmap0-eng.html and subnational divisions from The Humanitarian Data Exchange (OCHA) https://data.humdata.org/dataset/?vocab_Topics=administrative+boundaries-divisions.

Rhodesiense HAT cases in Estern and Southern Africa.
Period 2021–2022.

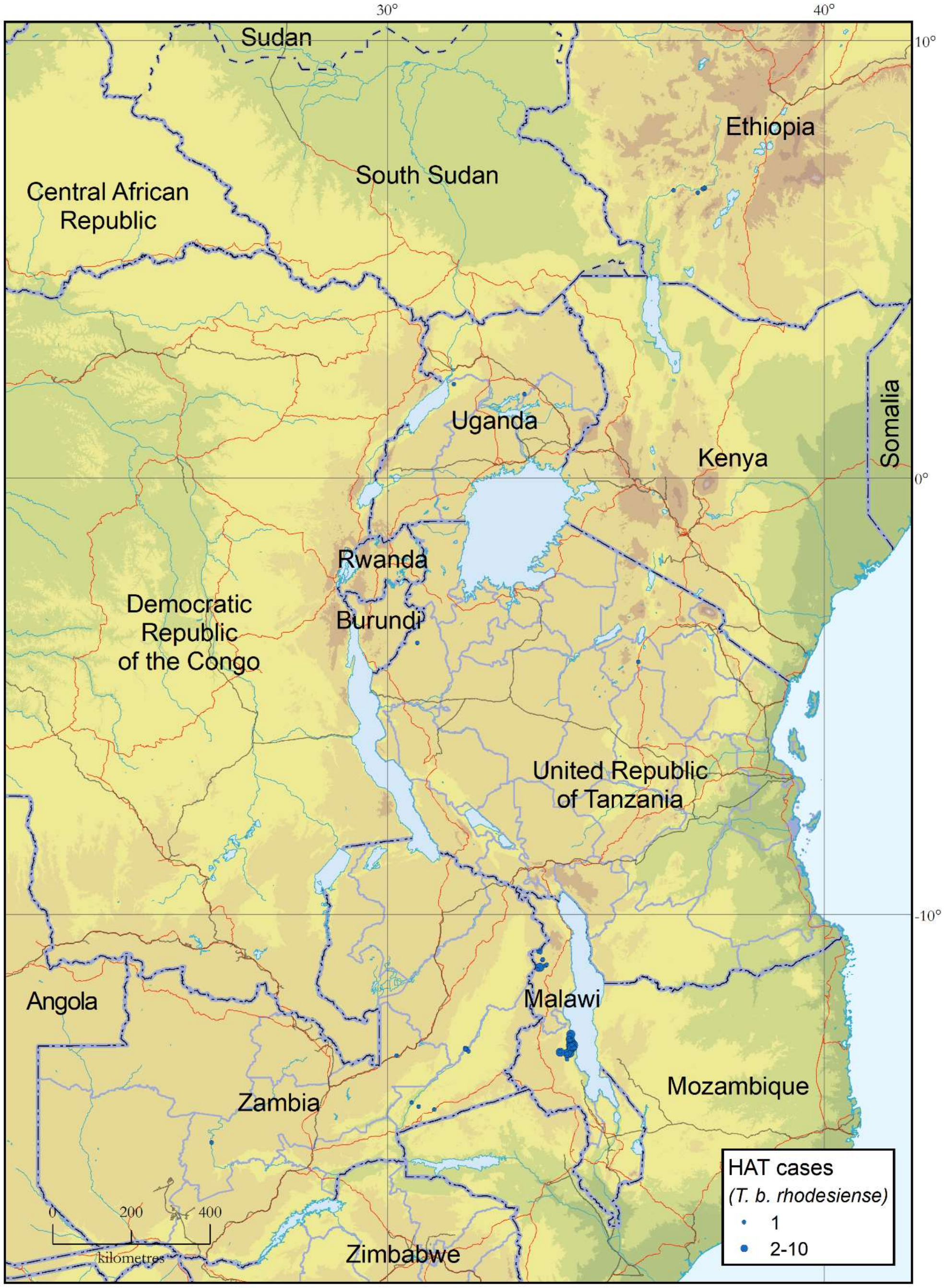

The base layers used in this map are the FAO Global Administrative Unit Layers (GAUL) https://data.apps.fao.org/map/catalog/srv/eng/catalog.search#/metadata/9c35ba10-5649-41c8-bdfc-eb78e9e65654, Shuttle Radar Topography Mission (SRTM) https://doi.org/10.5066/F7F76B1X, FAO Inland water bodies in Africa https://data.apps.fao.org/map/catalog/srv/eng/catalog.search;jsessionid=B7AF7A215B16770A1A67C65D97FF21CA?node=srv#/metadata/bd8def30-88fd-11da-a88f-000d939bc5d8, FAO Rivers of Africa https://data.apps.fao.org/map/catalog/srv/eng/catalog.search;jsessionid=B7AF7A215B16770A1A67C65D97FF21CA?node=srv#/metadata/b891ca64-4cd4-4efd-a7ca-b386e98d52e8, Vector Map Level 0 (VMap0) https://gis-lab.info/qa/vmap0-eng.html and subnational divisions from The Humanitarian Data Exchange (OCHA) https://data.humdata.org/dataset/?vocab_Topics=administrative+boundaries-divisions.
