# Supplementary material for: The elimination of human African trypanosomiasis: Monitoring progress towards the 2021–2030 WHO road map targets
Source: PLoS Negl Trop Dis. 2024 Apr 16;18(4):e0012111. doi: 10.1371/journal.pntd.0012111 (PMC11073784; doi:10.1371/journal.pntd.0012111)
Supplement: S2 Fig — Period 2016–2020 (PDF). The base layers used in the maps are the FAO Global Administrative Unit Layers (GAUL), Global Administrative Areas and FAO Inland water bodies in Africa. (PDF) [file pntd.0012111.s004.pdf]

Areas at risk of gambiense HAT infection in Western Africa. Period 2018–2022.

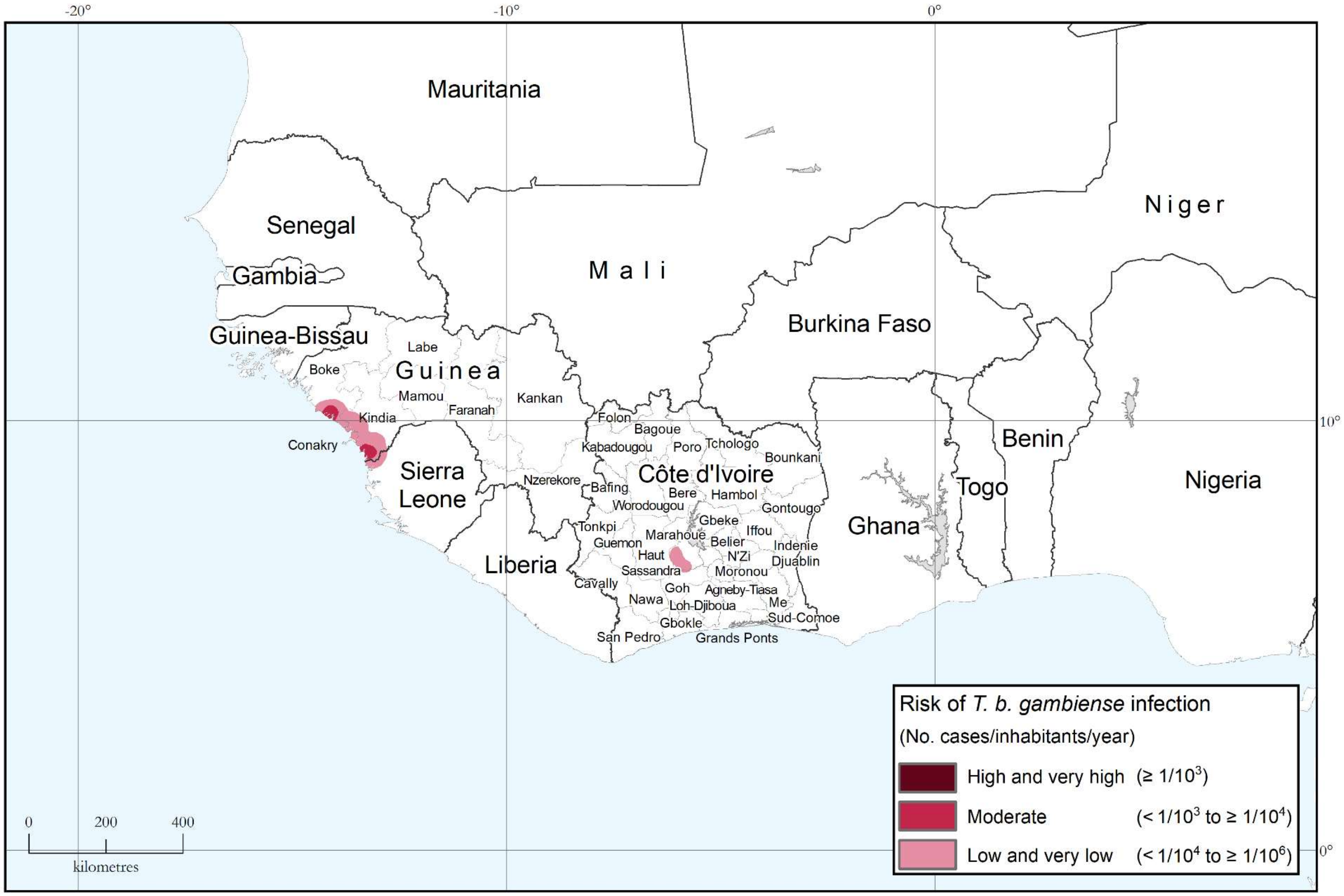

The base layers used in this map are the FAO Global Administrative Unit Layers (GAUL) https://data.apps.fao.org/map/catalog/srv/eng/catalog.search#/metadata/9c35ba10-5649-41c8-bdfc-eb78e9e65654, FAO Inland water bodies in Africa https://data.apps.fao.org/map/catalog/srv/eng/catalog.search;jsessionid=B7AF7A215B16770A1A67C65D97FF21CA?node=srv#/metadata/bd8def30-88fd-11da-a88f-000d939bc5d8 and subnational divisions from The Humanitarian Data Exchange (OCHA) https://data.humdata.org/dataset/?vocab_Topics=administrative+boundaries-divisions.

Areas at risk of gambiense HAT infection in Central Africa. Period 2018–2022.

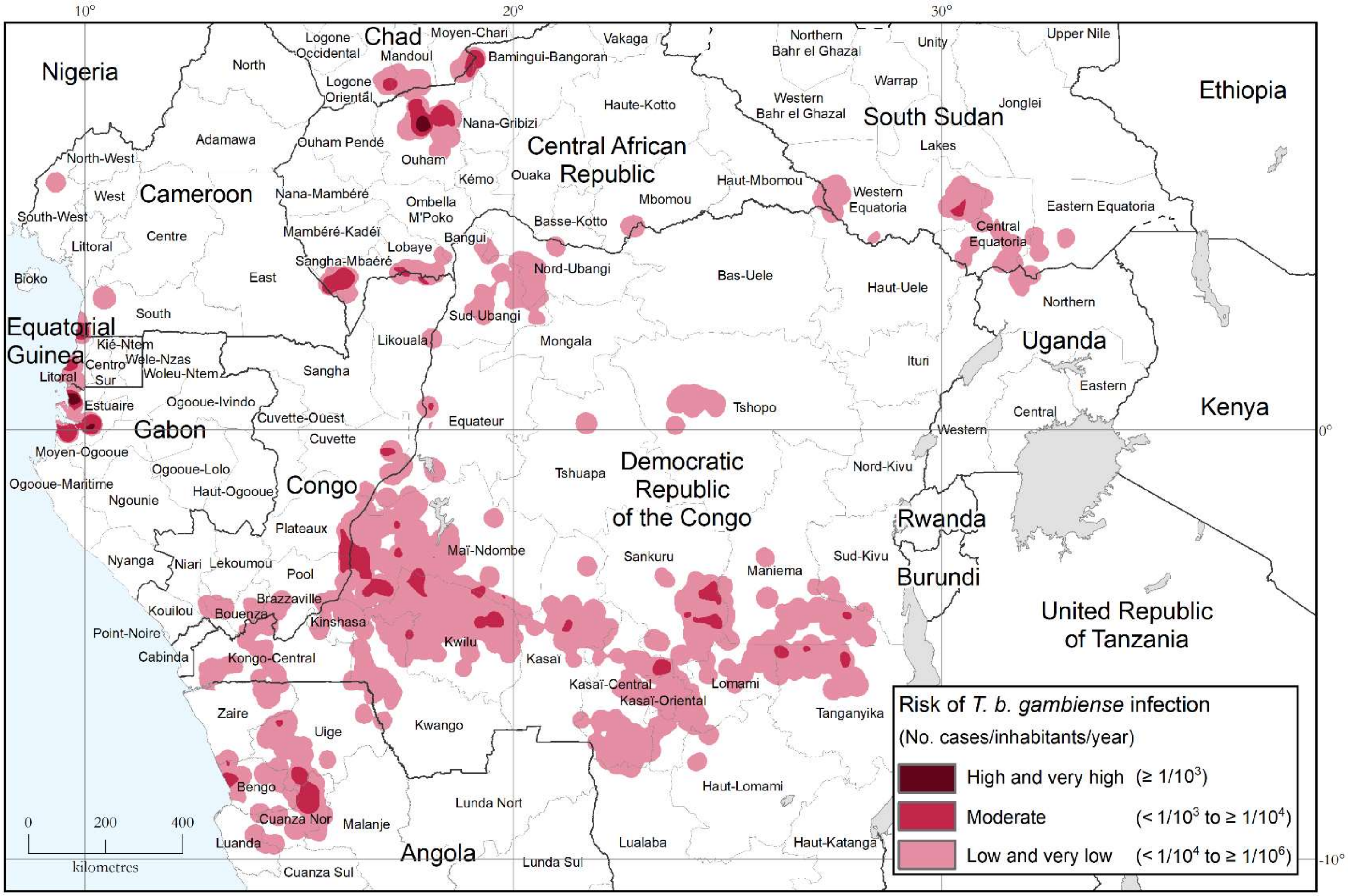

The base layers used in this map are the FAO Global Administrative Unit Layers (GAUL) https://data.apps.fao.org/map/catalog/srv/eng/catalog.search#/metadata/9c35ba10-5649-41c8-bdfc-eb78e9e65654, FAO Inland water bodies in Africa https://data.apps.fao.org/map/catalog/srv/eng/catalog.search;jsessionid=B7AF7A215B16770A1A67C65D97FF21CA?node=srv#/metadata/bd8def30-88fd-11da-a88f-000d939bc5d8 and subnational divisions from The Humanitarian Data Exchange (OCHA) https://data.humdata.org/dataset/?vocab_Topics=administrative+boundaries-divisions.

Areas at risk of rhodesiense HAT infection in Eastern and Southern Africa. Period 2018–2022.

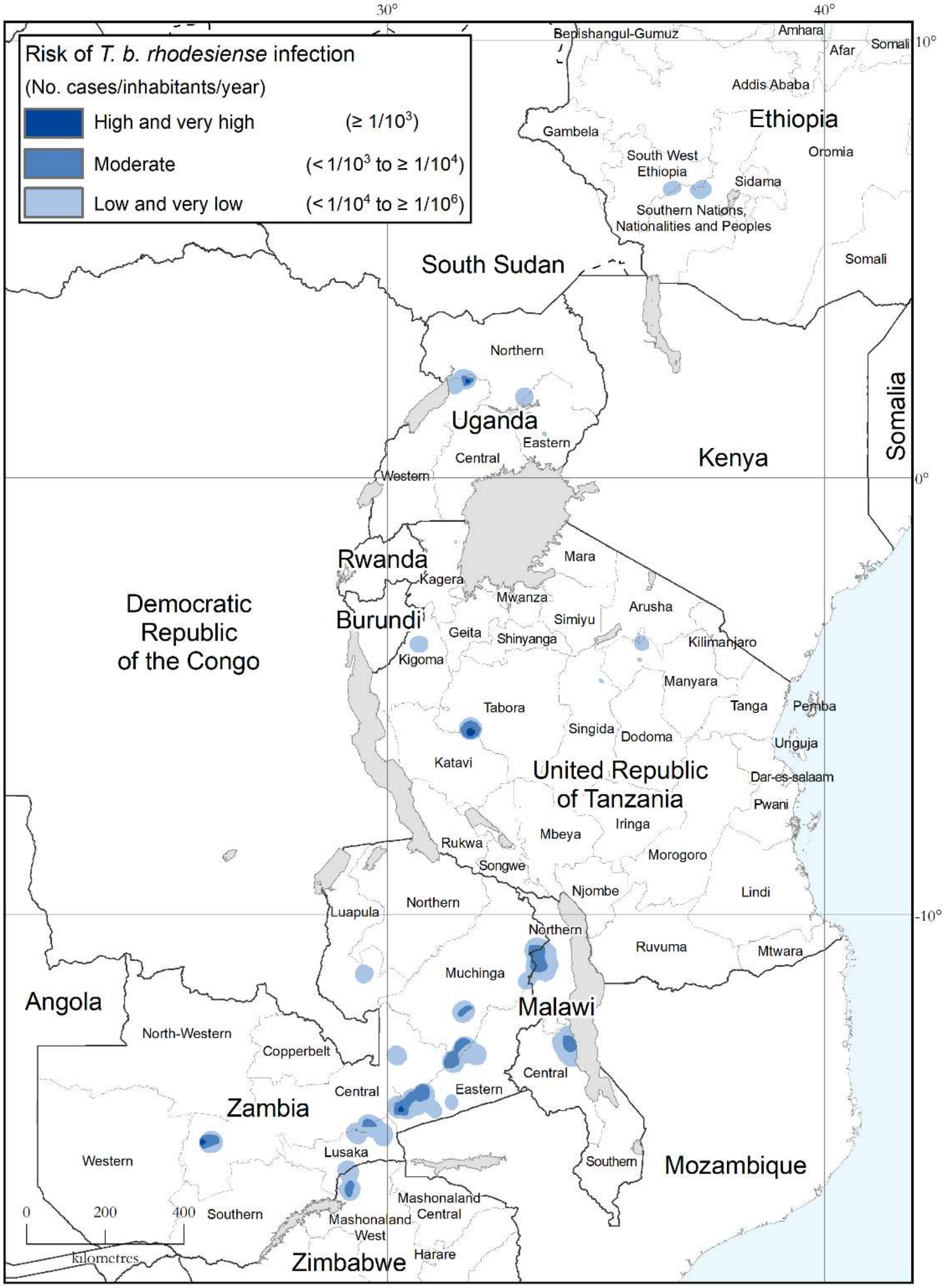

The base layers used in this map are the FAO Global Administrative Unit Layers (GAUL) https://data.apps.fao.org/map/catalog/srv/eng/catalog.search#/metadata/9c35ba10-5649-41c8-bdfc-eb78e9e65654, FAO Inland water bodies in Africa https://data.apps.fao.org/map/catalog/srv/eng/catalog.search;jsessionid=B7AF7A215B16770A1A67C65D97FF21CA?node=srv#/metadata/bd8def30-88fd-11da-a88f-000d939bc5d8 and subnational divisions from The Humanitarian Data Exchange (OCHA) https://data.humdata.org/dataset/?vocab_Topics=administrative+boundaries-divisions.
